# Effects of reduced chemical application by mechanical-chemical synergistic weeding on maize growth and yield in East China

**DOI:** 10.3389/fpls.2022.1024249

**Published:** 2022-09-26

**Authors:** Huimin Fang, Mengmeng Niu, Xinzhong Wang, Qingyi Zhang

**Affiliations:** ^1^ School of Agricultural Engineering, Jiangsu University, Zhenjiang, China; ^2^ Key Laboratory of Modern Agricultural Equipment and Technology (Jiangsu University), Ministry of Education, Zhenjiang, China; ^3^ Field operation technology and equipment innovation center, Shandong Academy of Agricultural Machinery Sciences, Jinan, China; ^4^ School of Mechanical Engineering, Shandong University, Jinan, China

**Keywords:** maize weed, alternative weeding practices, mechanical-chemical synergy, herbicide reduction, leaf area, dry matter weight, yield

## Abstract

There is growing concern about the environmental impact of chemicals and the long-term effects of mechanical weeding, which inhibits weed regrowth. Mechanical-chemical synergy has become an alternative weeding practice. In this paper, the effects of reduced chemical application by mechanical-chemical synergetic weeding on maize growth and yield are studied *via* synergistic weeding experiments. Experiments were carried out using three chemical reduction ratios (25%, 50%, and 75%) and two chemical applications (full width and only seeding row). The existing inter- and intra-implements were integrated as weeding machinery for full range mechanical weeding. Two indicators (leaf area and dry matter weight) were defined as growth characteristics at the filling and maturity stages. The results show that the leaf area of mechanical-chemical synergistic treatments was larger than those of single mechanical or chemical weeding treatments at the filling stage, but there was no significant difference between the leaf area values of the synergetic treatments (*P*=0.939). At the filling and maturity stages, the dry matter weight of mechanical-chemical weeding treatments was greater compared to the chemical weeding treatment. At the filling stage, the dry matter weight of the mechanical-chemical synergistic weeding treatments was less than that of the mechanical weeding treatment. In contrast, at the maturity stage, the dry matter weight of mechanical-chemical weeding treatments was greater, indicating that the promotional effect of the mechanical-chemical synergistic model was more pronounced at the later stage of crop growth. Single weeding or non-weeding treatment significantly affected the number of grains per ear (p=0.037) and 1000 grain weight (p=0.019), but it has been observed to have no significant effect on yield (p=0.504). The number of grains per ear, 1000 grain weight, and yield of the mechanical-chemical synergistic treatment were observed to be better than those of the chemical treatment. When compared with the full range of mechanical weeding treatments, only synergistic treatment produced a higher yield. From the perspectives of leaf area and dry matter, yield and its components, at the filling and maturity stage, the effect of mechanical-chemical synergy with 50% chemical reduction is the best recommendation as it reduces the dosage of chemical application, without significantly affecting crop growth and yield.

## 1 Introduction

Weeding is an important aspect of sustainable cropping. It is used to eliminate competitors for crop resources, facilitating nutrition and enabling access to sunlight, water, and space, which leads to improvements in yield and quality (Pannacci et al., 2017; Ahmad et al., 2020). Weed management is considered one of the most challenging tasks in crop production (Gharde et al., 2018; Chang et al., 2021) . Effective weed control can increase productivity per unit area (Qi et al., 2020). Improper weed management may cause a potential yield loss of approximately 32%, and this loss may continue to increase every year (Wang A. et al., 2019).

Common weed control methods include manual, mechanical, chemical, and more recent methods including electric shock, laser, and foam weeding, etc. Among them, manual (bare-handed or hand-held) weeding is convenient but labor-intensive, cumbersome, and time-consuming (Shrinivasa et al., 2017). The newly developed electric shock, laser, and foam weeding methods are not widely used due to their financial costs and relatively immature technology. Therefore, mechanical and chemical weeding are the two widely adopted weeding methods in field production. Mechanical weeding can quickly and effectively control weeds (Rao et al., 2018; Quan et al., 2021) and is, therefore, comparatively more widely used. Researchers have developed various weeding machines based on hoe shovel (Niu et al., 2022), claw tooth (Hu et al., 2012), rotary knife (Ma et al., 2022), finger (Pannacci et al., 2017; Machleb et al., 2021), and other key components to remove weeds between and within rows, such as Garford company’s Robocrop Inrow weeder and Kult Kress company’s finger weeder machine. Conventional mechanical weeding, which involves tillage, turning, raking, and other measures to cut/turn weeds out of the ground still lacks the ability to break the aggregated state of soil and weeds (Fang et al., 2022), failing to completely remove the weeds (Wang and Chen, 2017). Since the introduction of herbicides in the mid-20th century, chemical weeding has become more widely adopted (Hall et al., 2000). However, due to excessive dependence on chemical herbicides, a series of new problems including the emergence of resistant weed populations, the accelerated succession of weed communities, rapid development of weed resistance, and frequent occurrence of crop pests have introduced new challenges to sustainable weed management (Wang P. et al., 2019).

Integrated weed management (IWM) systems that combine non-chemical tactics with herbicides are becoming critical (Beam et al., 2021; Cordeau et al., 2022). Therefore, some researchers have developed integrated methods, combining mechanical and chemical weeding technology to reduce the use of herbicides while ensuring crop yield. Mulder and Doll (1993) proposed a weeding treatment method that combines herbicides with mechanical weeding and pointed out that the risk of yield loss was minimal when herbicide use was reduced by 50% to 75%. Heydel et al. (1999) adopted inter-row mechanical weeding combined with strip spraying to reduce herbicide dosage by 73%. Donald et al. (2001) combined inter-row mechanical weeding and reduced application of chemical herbicide for weeding of maize and soybean. The results showed that this method reduced herbicide use by 50%, with the weed level effectively controlled. Fang et al. (2022) studied the effects of inter-row and intra-row mechanical-chemical synergism from the perspective of weed control and crop growth. The results of the study showed that the mechanical-chemical synergism weeding mode could promote the accumulation of plant nutrient elements and crop growth.

Inter-row mechanical weeding methods are widely studied in research on mechanical-chemical weeding strategies. It should be noted that in addition to inter-row weeds, the intra-row weeds also significantly reduce the yield by 18-76% (Alba et al., 2020). The integrated inter- and intra-row weeding (IIIRW) system may be the future alternative (Kumar et al., 2020; Nsc et al., 2021). Additionally, existing studies tend to use yield as an evaluation index for crop growth, limiting an in-depth understanding of the impact of weeding strategies on crop growth, which makes the optimization of cooperative operation strategies difficult.

Considering the above-mentioned problems, this study introduced the leaf area and dry matter weight at grain filling stage during maize grain formation as an indicator to characterize the effects of different weeding treatments on plant growth and yield. The study tested the effects of synergistic weeding treatments on crop growth by utilizing the full range of mechanical weeding systems in combination with chemical weeding. The effect of synergistic weeding treatment was also compared to single weeding treatment based on the growth characteristics of maize at the filling and maturity stages. The experiments successfully optimized mechanical-chemical synergistic schemes by reducing herbicide application by 50%.

## 2 Materials and methods

### 2.1 Experimental site

The experiment was conducted on Zaoyuan Street, Zhangqiu District, Jinan City, Shandong Province from June to October 2021. The experimental field extends from latitude 36°25′ N to 37°09′ N and longitude 117°10′ E to 117°35′ E. The site locates in the warm temperate humid monsoon climate zone. The average annual amount of sunshine is 2647.6 h. The study site has an annual mean temperature of 12.8 °C and an annual precipitation of approximately 600.8 mm, with the frost-free period of about 192 days. The soil fertility of the test field was designated as medium fertile, the content of soil organic matter was tested to be 11.85 g/kg, alkali hydrolyzed nitrogen was 65.11 mg/kg, available phosphorus was 22.30 mg/kg, available potassium was 144.12 mg/kg, pH value was recorded at 8.18, and soil wet density was 1.43 g/cm^3^.

The maize cultivar Denghai 60, was used for the experiment and was sown on July 2, 2021. The planting mode was equal to row spacing of 70 cm and plant spacing of 22.5 cm. During the 4-5 leaf stage of maize, the occurrence of weeds in the field was high but relatively weak and easy to remove. Weeds, such as Pharbitis nil (L.) Ching, Digitaria sanguinalis, Eleusine indica, Portulaca oleracea, etc., are evenly distributed in the experimental site. The number and fresh weight of weeds per hectare are 95004 and 60596.4 g, respectively. It is the key period for weeding after seeding. At this time, the average soil moisture content in 0-5 cm and 5-10 cm soil layers was 16.29% and 16.45%, respectively, and the soil compactness at 5 cm and 10 cm was 0.71 MPa and 0.46 MPa, respectively.

### 2.2 Experimental weeding machinery and chemicals

**Figure 1:**
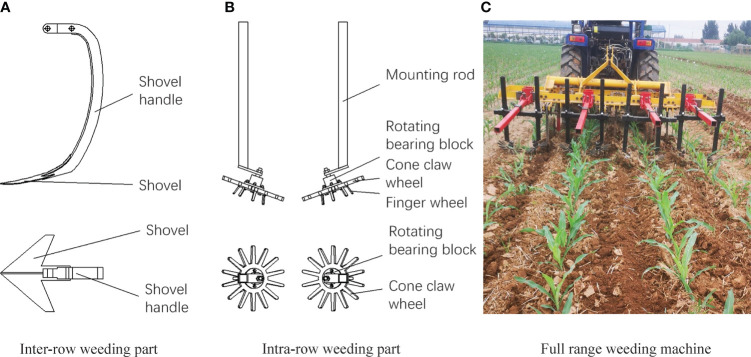
Experimental weeding parts and machine **(A)**, inter-row weeding part **(B)**, intra-row weeding part and **(C)**, full range weeding machine.

The mechanical weeding machines used in the experiment were independently developed. The inter-row weeding machine was of the hoe shovel type (**Figure 1A**), the intra-row weeding machine was of the finger type (**Figure 1B**), and the full range weeding machine was a combination of the two (**Figure 1C**). All of these weeding machines were mounted on a tractor as the power to draw the inter-row, intra-row, and full range weeder for mechanical weeding, and the operating speed was maintained at 3 km/h throughout the experiment duration. The herbicide used in the experiment was nicosulfuron· mesotrione· atrazine 24% oil dispersion agent (Shandong Province Jinan saipu Industrial Co., Ltd.), which was sprayed using a manual backpack electric sprayer (3WBD-20, from Taizhou Jiaojiang Lujia sprayer factory).

### 2.3 Experimental design

The experimental design was formulated by orthogonal experiment, taking into full consideration the single or synergistic weeding method, dosage of herbicide applied and spraying target. The detail of each experimental treatment is provided in **Table 1**. The treatments were randomly arranged and repeated three times, with each experimental plot of size 40 m^2^ (20 m long and 2 m wide). Post-sowing and pre-seedling herbicides were not used on any of the experimental plots, and no additional weeding treatment was applied after the test treatment.

**Table 1:** Experimental treatment.

Treatment	Mechanical weeding method	Application rate of herbicide (%)	Spraying range
Control T1	/	/	/
T2	/	100	Full width
T3	Full range	/	/
T4	Inter-row	/	/
T5	Intra-row	/	/
T6	Full range	75	Full width
T7	Full range	50	Full width
T8	Full range	25	Full width
T9	Full range	75	Seeding row
T10	Full range	50	Seeding row
T11	Full range	25	Seeding row

The non-weeding treatment of T1 was set as the control group where none of the pre and post-seedling herbicides or any other weeding treatments were applied during the whole maize growth period. The chemical weeding method used the common chemical agent nicosulfuron· mesotrione· atrazine 24% oil dispersion that is often used by the farmers, and the recommended dosage was 200 mL nitrate, tobacco, and atrazine 24% oil dispersion agent mixed in 20 L water for conventional spraying. 200, 150, 100, and 50 mL herbicide were mixed in 20 L water to represent the dosage of herbicide for 100%, 75%, 50%, and 25%, respectively. Mechanical weeding was carried out in three ways, inter-row, intra-row, and full range.

Mechanical-chemical synergistic weeding was carried out with mechanical weeding first, followed by chemical application. The dosage of chemical herbicides was decreased by 25%, 50%, and 75% on the basis of recommended dosage. The chemical spraying range of the synergistic weeding test was carried out in two ways: full-width application (conventional application, with the application area accounting for 100% of the whole plot) and seedling row application (only spraying crop rows, with the application area accounting for about 50% of the whole plot).

### 2.4 Experimental indicators and methods

#### 2.4.1 Crop leaf area

At the filling stage of maize, six consecutive plants with consistent growth were selected in each experimental plot, and length and width of the leaves were measured to calculate the leaf area of the plants. Leaf area of expanded leaves = maximum length × maximum width × 0.75 and Leaf area of unexpanded leaves = maximum length × maximum width × 0.5.

#### 2.4.2 Crop dry matter weight

At the filling stage and maturity stage of maize, six consecutive plants with consistent growth were selected in each experimental plot. The plants were desiccated at a temperature of 105 °C for 30 minutes and then dried at 70-80 °C for 36-48 h. The dried weight was called the dry matter weight of the plant. At the filling stage, the dry matter weight of plants and ears were measured. At the maturity stage, the dry matter weight of the plant, cob, and grains were measured.

#### 2.4.3 Crop yield

At the maturity stage of maize, three yield measuring parties (2 lines×5 m) were selected in each test plot. First, total panicle number  *N*_0_ and total panicle weight  *M*_0_ were recorded in each yield measuring party. We then removed 10 ears and ensured that the actual quality of the 10 ears was as close as possible to 10×*M*_0_/*N*_0_. We then threshed the 10 ears and the quantity was recorded as  *M*_1_ . Some grains were sampled, dried, and weighed, and the water content of grains *MC*_1_ was calculated. In the final step, the yield was calculated using the following formula,


$$Y=\left[10000\times \frac{N_{0}}{N\times L\times R_{s}}\times \frac{M_{1}}{10}\times \left(1-MC_{1}\right)\right]/\left(1000\times 0.86\right)$$

where, *N* is the number of lines selected for yield measuring; *L* is the length of the selected yield measuring party, m; *R*_*s*_ is the row space of maize, m; *N*_0_  is the total number of plant ears in the yield measuring party;  *M*_1_ is the grain quality of 10 ears after threshing; *MC*_1_ is the water content of the grain.

### 2.5 Data processing and analysis

SPSS statistics 23 was used for data analysis of variance, and LSD test was used for the significance of the difference between the processing treatments. Excel 2016 was used for data calculation and mapping.

## 3 Results and discussion

### 3.1 Variation of leaf area at filling stage

**Figure 2:**
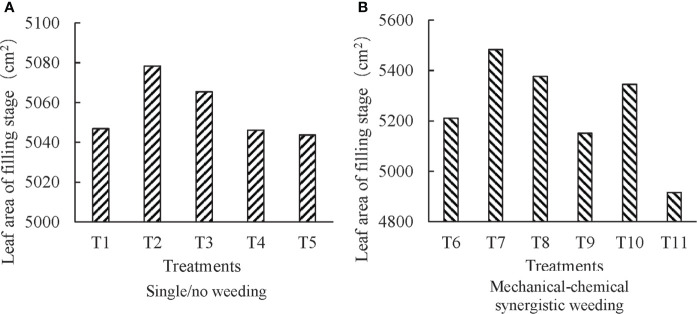
Effects of different weeding treatments on leaf area of filling stage **(A)**, single/no weeding treatments and **(B)**, mechanical-chemical synergistic weeding treatment.

Leaf area and leaf area index, which are closely related to photosynthesis and transpiration, are often used as characteristic parameters for monitoring crop growth and predicting crop yield (Lin et al., 2013). The values for these parameters within the threshold are deterministic of improved crop yield (James, 2004). The leaf area was selected to characterize the inhibitory or promotional effect of different experimental treatments on crop growth. The leaf area at the filling stage of the maize plant has been shown in **Figure 2**.

#### 3.1.1 Single weeding method

There was no significant statistical difference in leaf area between different treatments at the filling stage under several single weeding/non-weeding treatments (T1-T5) (*P* = 0.234). The highest leaf area was observed to be for the plants that underwent chemical weeding treatment (T2). Though the leaf area under chemical treatment (T2) was larger than that of three mechanical weeding (T3, T4, and T5) and non-weeding treatments (T1), the difference was statistically insignificant (*P* > 0.05). The leaf area of plants under chemical weeding treatment was significantly larger than that of mechanical weeding at the silking stage (Fang et al., 2022), but the difference at the filling stage was insignificant (P =0.100). This indicates that the initial inhibitory effect of weed regeneration on plant growth gradually disappeared from silking to the filling stage after a single mechanical weeding treatment. For three mechanical weeding methods, the statistical difference for leaf area of the plants that underwent treatments (full range > inter-row > intra-row) was insignificant (*P* > 0.05). The former experiment confirmed that the leaf area at the silking and maturity stage under the mechanical weeding treatment between rows was larger than that of the mechanical weeding between plants, but the difference was insignificant (Fang et al., 2022). Combined with the experimental results of the growing season at the grain filling stage, it is evident that the mechanical weeding treatment of inter-row, intra-row, and the combination of the two did not have any significant effect on the leaf area of growing plants.

#### 3.1.2 Synergistic weeding method

The leaf area was larger for the plants that had undergone chemical treatment in which herbicide application was reduced to 50% (full width and row application) in comparison to plants that received other reduction treatments. Our results also found that the leaf area of the full-width application treatment was larger than that of the row application treatment with the same dosage of herbicide application. However, there was no significant statistical difference between the leaf area values of the synergistic treatments (*P* = 0.939), indicating that these synergistic treatments did not have any significant effect on the leaf area of the plant at the filling stage.

With the exclusion of the treatment in which herbicide row application was reduced to 25% in T11 treatment, the leaf area of all mechanical-chemical synergistic treatments was greater than that of the single mechanical/chemical weeding treatment. Although the leaf area of the T11 treatment was smaller compared to the non-weeding/single weeding treatment, it was not significantly different from that of non-weeding (*P* = 0.795), chemical weeding (*P* = 0.748), and mechanical weeding (*P* = 0.767). This indicates that mechanical-chemical synergistic treatment can alleviate the stress of herbicides on crops. The integration of mechanical and chemical weeding is advantageous for crop growth. However, different synergistic patterns did not significantly affect the leaf area of plants at the filling stage.

### 3.2 Variation of dry matter weight at filling stage and maturity stages

**Figure 3:**
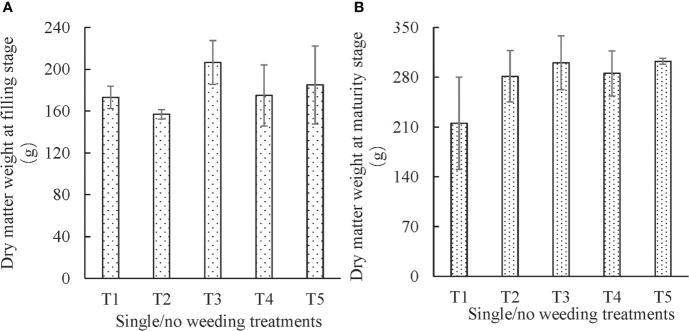
Effects of different single/no weeding treatments on dry matter weight of maize **(A)**, at filling stage and **(B)**, at maturity stage.

Dry matter is crucially related to crop yield formation, and its accumulation and distribution are proportional to the quality and quantity of crop yield (Wang et al., 2017). **Figure 3** provides details of the dry matter weight of maize at the filling and maturity stages under different experimental treatments.

#### 3.2.1 Single weeding method

The effect of no weeding/single weeding treatment was statistically insignificant on dry matter weight at the filling stage (*P* = 0.213) and maturity stage (*P* = 0.120). The dry matter weight at the filling stage was higher for plants that underwent mechanical weeding followed by non-weeding and chemical weeding. The dry matter weight at the maturity stage was higher for plants that had undergone mechanical weeding followed by chemical weeding and non-weeding. The dry matter weight at the filling and maturity stage indicates that mechanical weeding is a better treatment option to promote the accumulation of plant nutrient elements compared to non-weeding and chemical weeding. In particular, the dry matter of plants that had received the full range of mechanical weeding treatment (T3) at the filling stage was significantly higher than that of chemical weeding (T2) (*P* = 0.029). At the maturity stage, the dry matter of plants that had received full range of mechanical weeding treatment (T3) was significantly higher than that of non-weeding (T1) (*P* = 0.026). For the three mechanical weeding treatments (T3, T4, and T5), the dry matter weight of the plants that underwent a full range of mechanical weeding treatment (T3) and intra-row mechanical weeding treatment (T5) was the highest at the filling and maturity stage, respectively. The difference in dry matter weight between the full range (T3) and intra-row weeding treatment (T5) was not statistically significant at both filling and maturity stages. For the single mechanical weeding treatment, it can be inferred that the three mechanical weeding treatments had no significant effect on the leaf area of the plants at both filling and maturity stages.

The dry matter weight was observed to be low for the plants that had received chemical weeding treatment in terms of no weeding/single weeding treatments at the filling and maturity stages. The dry matter weight increased from 156.96 to 281.31 g from the filling to maturity stage, and the increment (124.35 g) and growth rate (79.22%) were the highest among the treatments. The dry matter weight of chemical weeding was 23.97% lower than that of full range mechanical weeding at the filling stage, and only 6.30% lower at the maturity stage, indicating that the chemical herbicide had an inhibitory effect on the growth of crops, which was much more pronounced at the later stage (i.e. the maturity stage). The dry matter of plants under non-weeding treatment only increased by 24.34% from the filling to maturity stage, indicating that the presence of weeds had an impact on the development of crops for the whole growth period.

In terms of the proportion of grain weight to dry matter weight of the whole plant, the values were highest for the full range mechanical weeding treatment (T3) at the maturity stage. The values of inter-row weeding (T4) and intra-row weeding (T5) treatments were higher than those of plants that received chemical weeding (T2) and non-weeding (T1) treatments. The difference between no weeding/single weeding treatment on the proportion of grain weight to dry matter weight of the whole plant was not statistically significant (*P* = 0.675). At the same time, each treatment had no significant effect on the proportion of grain weight to ear weight (*P* = 0.368), although the values of full range mechanical weeding treatment (T5) were higher than those of chemical (T2) and non-weeding (T1) treatments, and also higher than those of inter-row mechanical weeding (T4) and intra-row mechanical weeding treatment (T5).

#### 3.2.2 Synergistic weeding method

**Figure 4:**
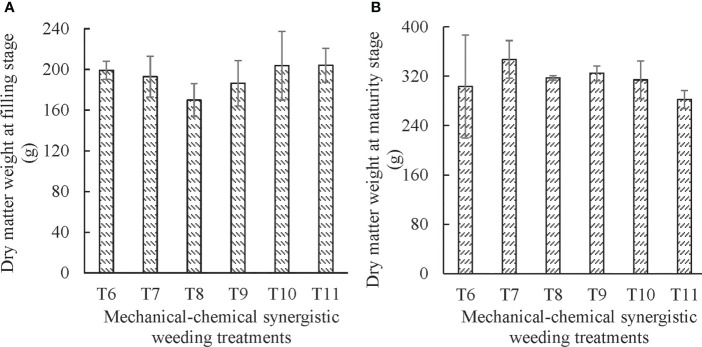
Effects of different synergistic weeding treatments on dry matter weight of maize **(A)**, at filling stage and **(B)**, at maturity stage.

The dry matter weight of maize at filling and maturity stages under different synergistic weeding treatments has been provided in **Figure 4**. The effects of mechanical-chemical synergistic weeding treatments on dry matter weight at the filling and maturity stage were insignificant.

The dry matter weight under the mechanical-chemical synergistic weeding treatment was higher as compared to chemical weeding treatment at the filling stage, and there were significant differences between T6 and T2 (*P* = 0.014), T7, and T2 (*P* = 0.032), T10 and T2 (*P* = 0.007), T11and T2 (*P* = 0.007). The results showed that the mechanical-chemical synergistic treatment was more beneficial for the accumulation of nutrient elements in plants than the single chemical treatment. The dry matter weight of the plants that underwent mechanical-chemical synergistic weeding treatment was less than that of single mechanical weeding treatment, which indicates that the chemical herbicide sprayed in a reduced dosage under the synergistic weeding treatment still has a certain inhibitory effect on crop growth, which can be confirmed from the minimum dry matter weight of plants that received single chemical treatment. The dry matter weight of plants that underwent herbicide reduction of 75% with spraying only rows treatment (T11) was the largest. This treatment (T11) was only significantly different from that of 75% herbicide reduction with spraying full width treatment (T8) (*P* = 0.040). It shows that the spraying target of herbicide will significantly affect the dry matter weight at the filling stage when the dosage of herbicide is reduced substantially.

At the maturity stage, the dry matter weight of mechanical-chemical synergistic weeding treatment was higher than that of plants receiving chemical weeding treatment. The dry matter weight of spraying only rows treatment (T11) was slightly less than that of mechanical weeding treatment. The dry matter weight of other synergistic treatments was greater than that of mechanical weeding (T3, T4, and T5). It indicates that the promotional effect of mechanical-chemical synergistic weeding treatment on crop growth was much more pronounced in the later stage of crop growth. At this stage, the inhibitory effect of chemical herbicides was minimized and mechanical weeding played its role to promote crop growth. At the maturity stage, the dry matter weight of full range spraying with 50% herbicide reduction treatment (T9) was the highest, which was 15.60% and 23.37% higher than the single mechanical weeding (T3) and single chemical weeding (T2), respectively.

From the filling to maturity stage, the increase in dry matter weight reached 154.19 g under T7 treatment, and grain weight constituted 34.32% of dry matter weight. The growth rate of dry matter weight under T8 treatment was the highest, reaching 86.73%, of which 35.26% was grain weight. The treatment with the lowest growth amount and growth rate of dry matter weight occurred in rows that had only been sprayed with the herbicide reduction of 75% (T11). Under this treatment, the growth amount was 40.53% lower than the average growth amount of all synergistic treatments, and the growth rate was 36.09% lower than the average. It showed that a considerable reduction in herbicide application on the row affects the accumulation of dry matter. The proportion of grain weight to the dry matter weight of the whole plant at the maturity stage was higher for all other synergistic treatments than that of the chemical weeding treatment. The proportion of 25% (T9) and 50% reduction (T10) treatments of herbicide proportion applied to the rows were significantly higher (*P* = 0.009 and *P* = 0.010, respectively) than that of the chemical treatment (T2).

### 3.3 Yield and its components

The maize yield and its components under different weeding treatments were provided in **Table 2**.

**Table 2:** Maize yield and its components under different treatments.

Weeding method	Treatment	Yield/(kg·hm-2)	Grain number per ear	1000-grain weight/g
Single/no weeding	T1	6918.5 ± 2089.53 d	539.8 ± 81.45 cd	304.07 ± 31.43 abd
	T2	7453.34 ± 1684.64 cd	525.4 ± 72.66 d	267.29 ± 42.63 f
	T3	8792.89 ± 840.84 abc	626.6 ± 82.31 a	313.81 ± 25.42 ab
	T4	8226.74 ± 848.6 bcd	558.4 ± 56.3 bcd	280.16 ± 37.38 def
	T5	8179.77 ± 622.51 bcd	558.2 ± 70.68 bcd	313.52 ± 32.77 ab
Mechanical-chemical synergistic weeding	T6	9344.98 ± 591.07 ab	592 ± 68.92 abc	285.34 ± 27.43 cdef
	T7	9233.56 ± 917.58 ab	606.8 ± 63.61 ab	296.38 ± 22.37 bcde
	T8	8678.5 ± 313.23 abc	578.8 ± 48.49 abcd	285.47 ± 6.76 cdef
	T9	10063.01 ± 541.71 a	587.8 ± 51.02 abc	306.04 ± 15.11 abc
	T10	9235.52 ± 771.4 ab	557.4 ± 73.04 bcd	323.47 ± 13.6 a
	T11	7760.47 ± 731.2 bcd	614.8 ± 56.88 ab	276.29 ± 11.33 ef

#### 3.3.1 Effect of weeding method

The maize yield under single weeding/no weeding treatment was higher for the full range mechanical weeding (T3) followed by inter-row mechanical weeding (T4), intra-row mechanical weeding (T5), chemical weeding (T2), and non-weeding (T1), but there was no significant statistical difference between them (*P* = 0.504). However, the single weeding/non-weeding treatment significantly affected the number of grains per ear (*P* = 0.037) and the 1000 grain weight (*P* = 0.019). The number of grains per ear, 1000 grain weight, and yield under the full range mechanical weeding treatment (T3) were the highest, and there were significant differences between the number of grains per ear and other treatments (*P*< 0.05). The 1000 grain weight was only significantly higher for the full range mechanical weeding treatment (T3) than that of chemical weeding (T2) (*P* = 0.007) and inter-row mechanical weeding (T4) (*P* = 0.045). As far as the three mechanical weeding methods are concerned, the full range mechanical weeding treatment (T3) was observed to be advantageous in terms of the number of grains per ear, 1000 grain weight, and yield. Among the treatments (T3, T4, and T5), the number of grains per ear was significantly different from the inter-row mechanical weeding (T4) (*P* = 0.043) and the intra-row mechanical weeding (T5) (*P* = 0.043), and the 1000 grain weight was only significantly different for the inter-row mechanical weeding (T4) (*P* = 0.045). In terms of final yield, the full range mechanical weeding treatment (T3) produced a yield 6.88% higher than that of inter-row mechanical weeding treatment (T4) and 7.50% higher than the intra-row mechanical weeding treatment (T5), and no statistically significant difference was observed (*P* =0.590).

In terms of grain number per ear, 1000 grain weight, and yield, mechanical-chemical synergistic treatment was observed to be better than chemical weeding treatment. With the exclusion of full range spraying with 75% herbicide reduction treatment (T8) (*P* = 0.081), the number of grains per ear under the mechanical-chemical synergistic treatment was significantly higher than that of the chemical treatment (T2) (*P*< 0.05). The 1000 grain weight of T7, T9, and T10 treatments were significantly higher than that of chemical treatment (T2) (*P*< 0.05). In terms of yield, only the treatments T6 and T9 with 25% herbicide reduction produced significantly higher yield as compared to chemical treatment (T2) (*P*< 0.05). Compared with the full range mechanical weeding treatment, the mechanical-chemical synergistic treatment was only advantageous in producing a higher yield, except for T8 and T11 with 75% herbicide reduction. The yield of other synergistic treatments was higher as compared to full range mechanical weeding treatment (T3), but there was no significant difference between them (*P* > 0.05). The average yield of each mechanical-chemical synergistic weeding treatment was 21.46% and 2.95% higher than that of single chemical weeding treatment (T2) and full range mechanical weeding treatment (T3), respectively. The yield of treatment T9 with 25% herbicide reduction and sprayed only rows was 35.01% and 14.44% higher than that of single chemical weeding (T2) and full range mechanical weeding (T3), respectively.

#### 3.3.2 Effect of spraying target

In terms of spraying target, varying the dosage of herbicide application applied to the row only significantly affected the 1000 grain weight (*P* = 0.000) and crop yield (*P* = 0.017), but had no significant effect on the number of grains per ear (*P* = 0.129). The 1000 grain weight decreased significantly when the application rate decreased from 50% to 25% (*P* = 0.000). There was no significant effect on yield when the application rate was reduced from 75% to 50% (*P* = 0.192), but the effect is observed to be significant only when the application rate was further reduced. It shows that the dosage of herbicide will have a significant impact on the yield only when it is reduced to a certain dosage when the herbicide was applied to the row, which is consistent with the test results.

The effects of full width application on grain number per ear (*P* = 0.596), 1000 grain weight (*P* = 0.446) and yield (*P* = 0.459) were not significant. The number of grains per ear, 1000 grain weight, and yield values all were observed to be similar for both 25% and 50% reductions in full width herbicide spraying treatments. The number of grains per ear and 1000 grain weight were higher under 50% herbicide reduction treatment (T7) while the yield produced was observed to be higher for the 25% herbicide reduction treatment (T6) with full width spraying treatment.

#### 3.3.3 Effect of mechanical-chemical synergistic treatment

The mechanical-chemical synergistic treatments had no significant effect on the number of grains per ear (*P* = 0.358), but significantly affected the 1000 grain weight (*P* = 0.000) and yield (*P* = 0.024). The 1000 grain weight was significantly higher for 50% herbicide application spraying only rows treatment (T10) than that of all synergistic treatments. The 1000 grain weight of 75% herbicide application spraying full width treatment (T6) was significantly higher than that of 75% spraying only rows treatment (T9) (*P* = 0.016) and 50% spraying only rows treatment (T10) (*P* = 0.000). The 1000 grain weight of 50% herbicide application spraying full width treatment (T7) was significantly higher than that of 50% spraying only rows treatment (T10) (*P* = 0.002) and 25% spraying only rows treatment (T11) (*P* = 0.019). However, the 1000 grain weight of 25% herbicide application spraying full width treatment (T8) was not significantly higher than that of 25% spraying only rows treatment (T11) (*P* = 0.272). This shows that when the herbicide application is reduced by 25% and 50%, the effect of full width spraying is significantly better than that of row spraying. However, when the herbicide is reduced to a certain dosage (75%), the effect of full width spraying and row spraying on 1000 grain weight is no longer significant.

In terms of the yield produced, it decreases with the decrease in the dosage of herbicide. The highest yield was obtained under row spraying 75% treatment (T9), but it was only significantly higher than those of T8 (*P* = 0.027) and T11 (*P* = 0.001) of herbicide spraying 25%, indicating that the herbicide reduction of 25% or 50% did not have a significant impact on the yield. The crop yield was significantly affected only when the dosage of herbicide was reduced to 25%. The lowest yield was produced under 25% herbicide spraying on the row treatment (T11), except that there was no significant difference with T8 treatment, which was significantly lower than that of 75% and 50% herbicide spraying on the row or full width. Therefore, reducing herbicide application by 25% or 50% will not significantly affect the number of grains per ear, 1000 grain weight, and yield of crops.

### 3.4 The Recommended Mechanical-chemical Synergistic weeding Treatment

The experiment showed that, as far as the yield and its components were concerned, the inter-row/intra-row mechanical-chemical synergistic weeding treatment with a 25% reduction was beneficial for crop growth (Fang et al., 2022). Full range mechanical weeding combines the advantages of mechanical weeding between inter-row and intra-row weeding, and comprehensively removes inter and intra-rows weeds. This full range mechanical weeding can replace some herbicides to a certain extent. Under the premise of this full range mechanical weeding treatment, the herbicide application reduction of 25% or 50% can be used as the recommended treatment of herbicide application for mechanical-chemical synergistic weeding treatment.

Furthermore, in terms of leaf area and dry matter weight at the filling and maturity stage, the mechanical-chemical herbicide reduction treatment of 50% performed better than other treatments. Therefore, in order to minimize the application of herbicides, while not damaging crop growth and not affecting final yield, the mechanical-chemical synergistic effect of 50% herbicide reduction is the best recommended weeding treatment. The long-term positioning test can be conducted to verify its effectiveness.

## 4 Conclusion

In order to verify the effect of mechanical-chemical synergistic weeding on maize growth and provide a treatment reference for the full range mechanical-chemical synergistic weeding treatment, this paper introduced the leaf area and dry matter at grain filling stage during maize grain formation as an indicator to characterize the effects of different weeding treatments on plant growth and yields. With the exclusion of the treatment in which herbicide row application was reduced to 25% in T11 treatment, the leaf area of all mechanical-chemical synergistic treatments was greater than that of single mechanical/chemical weeding treatment. This indicates that mechanical-chemical synergistic treatment can alleviate the stress of herbicides on crops, and the integration of mechanical and chemical weeding is advantageous for crop growth. During the filling stage, the dry matter weight of the plants that underwent mechanical-chemical synergistic weeding treatment was less under the single mechanical weeding treatment, which indicates that the chemical herbicide sprayed in a reduced dosage under the synergistic weeding treatment still has a certain inhibitory effect on crop growth. At the maturity stage, with the exclusion of the treatment T11, the dry matter weight of other synergistic treatments was greater than that of mechanical weeding. This indicates that the promotional effect of mechanical-chemical synergistic weeding treatment on crop growth was much more pronounced in the later stage of crop growth. In terms of grain number per ear, 1000 grain weight, and yield, the mechanical-chemical synergistic treatment was observed to be better than chemical weeding treatment. Compared with the full range mechanical weeding treatment, the mechanical-chemical synergistic treatment was only advantageous to produce higher yield. Reducing herbicide application by 25% or 50% will not significantly affect the number of grains per ear, 1000 grain weight, and yield of crops. In terms of leaf area and dry matter weight at the filling and maturity stage, the mechanical-chemical herbicide reduction treatment of 50% performed better than other treatments.

## Data availability statement

The original contributions presented in the study are included in the article/supplementary material. Further inquiries can be directed to the corresponding authors.

## Author contributions

HF performed most of the experiments with the assistance of MN and QZ. HF, and QZ designed the study, analyzed the data, and wrote the manuscript. HF, QZ, and XW revised the manuscript. All authors contributed to the article and approved the submitted version.

## Funding

This research was funded by the National Natural Science Foundation of China (52005310), the Priority Academic Program Development of Jiangsu Higher Education Institutions (PAPD-2018-87), the China Postdoctoral Science Foundation (2021M701800), Jiangsu Postdoctoral Research Funding Program(2021K124B), and Open Project of Key Laboratory of Modern Agricultural Equipment, Ministry of Agriculture and Rural Affairs (2020007)

## Conflict of interest

The authors declare that the research was conducted in the absence of any commercial or financial relationships that could be construed as a potential conflict of interest.

## Publisher’s note

All claims expressed in this article are solely those of the authors and do not necessarily represent those of their affiliated organizations, or those of the publisher, the editors and the reviewers. Any product that may be evaluated in this article, or claim that may be made by its manufacturer, is not guaranteed or endorsed by the publisher.
